# The Genomics of Opioid Addiction Longitudinal Study (GOALS): study design for a prospective evaluation of genetic and non-genetic factors for development of and recovery from opioid use disorder

**DOI:** 10.1186/s12920-020-00837-3

**Published:** 2021-01-07

**Authors:** Jessica Heil, Stefan Zajic, Emily Albertson, Andrew Brangan, Iris Jones, Wendy Roberts, Michael Sabia, Elliot Bodofsky, Alissa Resch, Rachel Rafeq, Rachel Haroz, Russell Buono, Thomas N. Ferraro, Laura Scheinfeldt, Matthew Salzman, Kaitlan Baston

**Affiliations:** 1 Coriell Institute for Medical Research, 403 Haddon Ave, Camden, NJ 08103 USA; 2 Cooper University Health Care, 1 Cooper Plaza, Camden, NJ 08103 USA; 3 Rowan University, 201 Mullica Hill Rd, Glassboro, NJ 08028 USA

**Keywords:** Opioid use disorder (OUD), Genetics, Medication for opioid use disorder (MOUD), Addiction, Substance misuse, Buprenorphine

## Abstract

**Background:**

The opioid use disorder and overdose crisis in the United States affects public health as well as social and economic welfare. While several genetic and non-genetic risk factors for opioid use disorder have been identified, many of the genetic associations have not been independently replicated, and it is not well understood how these factors interact. This study is designed to evaluate relationships among these factors prospectively to develop future interventions to help prevent or treat opioid use disorder.

**Methods:**

The Genomics of Opioid Addiction Longitudinal Study (GOALS) is a prospective observational study assessing the interplay of genetic and non-genetic by collecting comprehensive genetic and non-genetic information on 400 participants receiving medication for opioid use disorder. Participants will be assessed at four time points over 1 year. A saliva sample will be collected for large-scale genetic data analyses. Non-genetic assessments include validated surveys measuring addiction severity, depression, anxiety, and adverse childhood experiences, as well as treatment outcomes such as urine toxicology results, visit frequency, and number of pre and post-treatment overdoses extracted from electronic medical records.

**Discussion:**

We will use these complex data to investigate the relative contributions of genetic and non-genetic risk factors to opioid use disorder and related treatment outcomes.

## Background

Millions of Americans each year take prescribed opioid analgesics, and the rate of non-medical use of prescription opioids has dramatically risen over the past decade. The majority of people who take opioids short term for pain relief do not develop opioid use disorder (OUD) [1], but for the unfortunate minority, these prescribed medications may lead to dependence. Opioid use disorder, also known as opioid addiction, occurs when a person progresses from physiological dependence and/or physiological tolerance to either prescribed or illicit opioids into opioids use interfering with daily life [2]. While population studies have demonstrated an inherent risk for developing substance use disorder based on genetic [3, 4] and non-genetic factors [5], the underlying question of why some develop an OUD and others do not is largely unresolved.

The current opioid epidemic has developed through several phases over the last 30 years [6]. Starting in the 1990s, there was a large increase in opioid prescriptions nationwide [7]. Prescription rates peaked in 2010 with 81.2 opioid prescriptions for every 100 Americans [8]. Following this increase in prescription rates, the production of even more powerful opioids such as non-pharmaceutical fentanyl (NPF) and other synthetic opioids accelerated the rate of OUD and opioid-related overdose death by 2013 [9, 10]. Between 1999 and 2014, the United States saw a three-fold increase in opioid-related overdose deaths [11]. This harrowing trend resulted in an estimated 130 opioid-related overdose deaths on average per day in 2017 [12]. Finally, the sudden restrictions placed on opioid prescriptions have pushed people out of desperation to turn to illicit opioid use (heroin, fentanyl, etc.) [13]. Altogether, it was estimated in 2017 that nearly 1% (2.1 million) of Americans over the age of 12 had an OUD, with the majority (80%) of active heroin addictions starting with the misuse of opioid prescriptions [14]. These successive phases of opioid misuse have expanded the scope of the crisis.

A family history of substance use disorder may demonstrate environmental risk for OUD but also suggests the potential for inherited, genetic risk factors [15–17]. The majority of published papers that examine inherited risk for OUD have not identified consistent genetic risk factors [18–21]. Reasons for this discrepancy include limited sample size, inconsistent analyses, and limited population control cohorts. More recently, a large genome-wide association study for OUD was published that includes an OUD cohort (*n* = 4503), a non-OUD, opioid exposed cohort (*n* = 4173), and a non-OUD, non-opioid exposed cohort (*n* = 32,500). Even with these relatively large cohort sizes, there were no statistically significant genetic differences between the OUD and non-OUD exposed groups [22]. Taken together, this body of work is consistent with a relatively complex and polygenic risk underlying the inherited component of OUD. In addition to genetic factors, OUD risk (or other opioid-related phenotypes) may also involve epigenetic mechanisms. Research in this area has thus far been limited to a handful of candidate gene studies (e.g. for OPRM1 [23, 24]) and one relatively small epigenome-wide association study [25].

Environmental risk factors are known to play an important role in OUD. Environmental risk factors for OUD include a range of psychological and social determinants of health [5]. Critically, these risk factors rarely occur in isolation and are not mutually exclusive in their contributions to the development of an OUD. Rates of mood and anxiety disorders (e.g. major depressive disorder, bipolar disorder, panic disorder) are disproportionately high in OUD populations [26] which suggests a self-medication pathway, with symptoms driving opioid misuse to mitigate emotional pain and heightened physiological arousal [27, 28]. Further, adverse childhood experiences (ACEs) such as parental substance use, physical or sexual abuse, and emotional neglect also serve as precursors to OUD [29]. These adverse childhood experiences undermine personal safety and stability and results in increased chronic stress, which can leave some more likely to engage unhealthy coping mechanisms [29, 30]. More generally, a lack of family and/or social support, either in childhood or adulthood, results in higher risk for substance misuse, especially when it occurs in tandem with psychological disorders and distress [31].

The opioid epidemic continues to shift and affect a growing number of families throughout the US, and the need for a more comprehensive understanding of the genetic and environmental factors that affect development and recovery from OUD is critical. New Jersey has felt the brunt of the opioid epidemic with higher than average opioid-related overdose mortality rates. In 2018, the state of New Jersey sustained 35.1 opioid-related deaths per 100,000 persons, a rate that is over 50% higher than the national average [32, 33]. Moreover, Camden County only accounts for 5.7% of New Jersey’s total population; however, the number of emergency naloxone (an emergency treatment to reverse opioid intoxication or overdose) doses administered in 2018 accounted for 20% of the total number of naloxone doses administered in the entire state of New Jersey [33].

With this goal in mind, we have launched the Genomics of Opioid Addiction Longitudinal Study (GOALS). Here, we describe the GOALS study design for collecting detailed demographic, clinical, and genetic information to investigate the development of OUD and, the outcomes of treatments for OUD over time.

## Methods/design

### Project overview

**Fig. 1:**
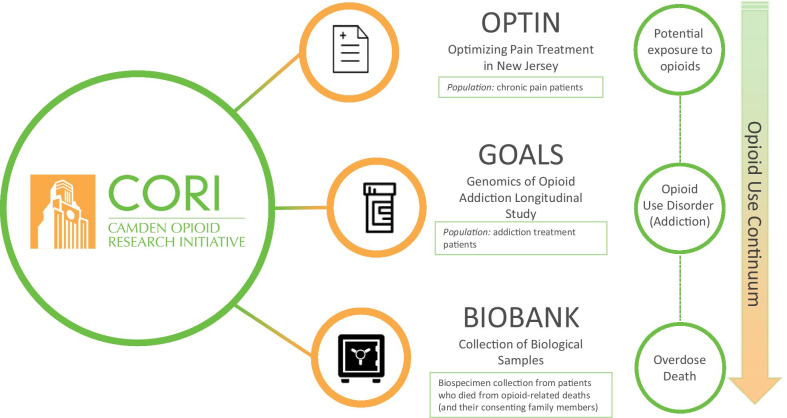
Overview of Camden Opioid Research Initiative and the Opioid Use Continuum

The Genomics of Opioid Addiction Longitudinal Study (GOALS) represents one arm of the Camden Opioid Research Initiative (CORI), a three-armed research project (Fig. 1) spanning the opioid use epidemic continuum. The three arms consist of an observational study evaluating treatment of chronic pain (Optimizing Pain Treatment in New Jersey (OPTIN)), a prospective observational study of participants in active treatment of OUD (GOALS), and creation of a genetic biobank of samples from individuals who have died from an opioid overdose (CORI Biobank). CORI is a collaborative research effort between the Coriell Institute for Medical Research, Cooper University Health Care, and Cooper Medical School of Rowan University. By collecting sets of detailed phenotypic and genetic information across the spectrum of opioid use from a diverse, understudied and underfunded patient population in Southern New Jersey, we are establishing a unique collection of biospecimens and associated data to evaluate factors contributing to OUD and related outcomes.

### Study design

**Fig. 2:**
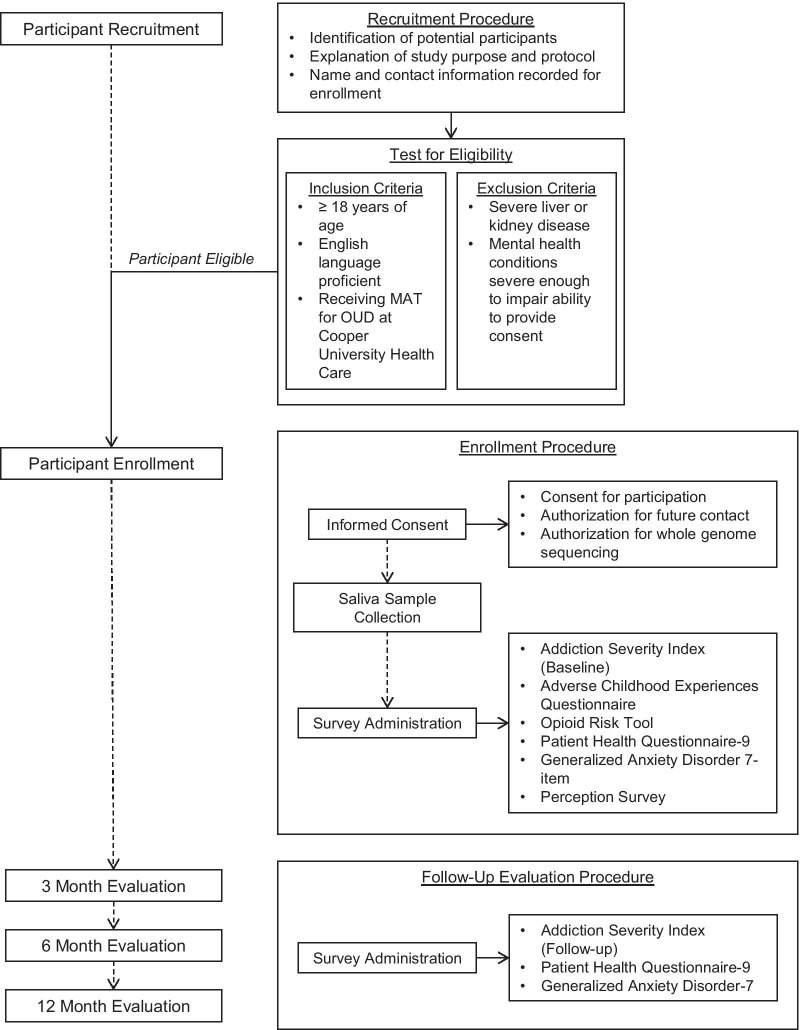
GOALS study visits schedule and assessments

This is a prospective, observational study evaluating genetic and non-genetic risk factors for OUD and medication for opioid use disorder (MOUD) related outcomes. This study is longitudinal with planned data collection at baseline, 3 months, 6 months, and 12 months post-baseline (Fig. 2). At baseline, participants are asked to provide a saliva sample for genetic testing and to complete questionnaires (Table 1) including questions about their mental health, childhood experiences, current use of opioids and other drugs, family and personal history of substance use, employment and legal issues. In addition to completing these surveys, participants are requested to permit access to electronic health records for 12 months post-baseline.

**Table 1:** Description of clinically validated GOALS surveys

Measure	Clinical Utility
Patient Baseline Perception Survey	A self-report survey authored by the research team, designed to gauge how much participants believe genetic and non-genetic (e.g. mental illness, traumatic events, age of first use, access/exposure to substances of abuse) factors contributed to their development of OUD.
Generalized Anxiety Disorder 7-item (GAD-7) Scale Baseline	A self-report scale designed to detect symptoms of Generalized Anxiety Disorders (GAD) as defined by the Diagnostic and Statistical Manual of Mental Disorders (4th edition; DSM-IV) and their severity. Increased scores on the GAD-7 are strongly associated with a variety of negative symptoms and behavioral outcomes, some of which have been empirically linked to specific patterns of substance use [34].
Patient Health Questionnaire (PHQ-9) Baseline	A self-report instrument that evaluates the presence and severity of depression symptoms as defined by the nine DSM-IV criteria used to screen for depressive disorders. In a substance-using population, higher PHQ-9 scores specifically indicate depression-proneness that may motivate this population to use substances that help regulate or suppress negative affect [35].
Opioid Risk Tool (ORT)	A brief self-report tool designed to estimate the likelihood of misusing prescription opioids with high specificity and sensitivity. Risk is assessed based on the presence/absence of five elements of an individual’s life: family history of substance abuse, personal history of substance abuse, age, history of preadolescent sexual abuse, and psychological diseases [36].
Adverse Childhood Experience (ACE) Questionnaire	A self-report measure designed to evaluate one’s history of adverse childhood experiences (ACEs) such as abuse, neglect, lack of social support, witnessing abuse, or dysfunctional family dynamics. ACE scores are positively correlated with the likelihood of overdose [37].
Addiction Severity Index (ASI)	Standard tool used for substance use evaluation around the state of New Jersey with interview-style administration. This tool captures details from seven areas of the individual’s life: general information, medical history, employment/support status, alcohol/drug history, legal history, family/social relationships, and psychiatric evaluation [38].

The Center for Healing at Cooper University Health Care is a low barrier specialty clinic for patients struggling with substance use disorders in Camden, New Jersey. This clinic has various satellite locations throughout southern New Jersey and is part of an integrated pain, behavioral health, and substance use disorder treatment team that includes emergency, hospital-based, and ambulatory care. The clinic staff is a multi-disciplinary team comprised of Addiction Medicine board-certified physicians, nurse practitioners and physician assistants as well as therapists, counselors, patient navigators, nurses, a pharmacist, and medical assistants all dedicated to providing care for this vulnerable patient population.

GOALS participants are recruited from the Center for Healing at Cooper University Health Care in Camden City that serves Southern New Jersey residents. The target sample size for this study is 400 participants, recruited over 2 years. Participants are provided with information about the study in individual or group settings by clinical research coordinators, as well as by their medical providers and other staff. Participants are able to indicate interest in participating in the study by speaking with coordinators in person, by phone, or by email. Perinatal patients are eligible for inclusion in this study. Coordinators evaluate interested participants for eligibility based on the following inclusion and exclusion criteria:

- Inclusion criteria
  - 18 years or older
  - English language proficient
  - Receiving MOUD for OUD treatment at Cooper University Health Care
- Exclusion criteria
  - Severe liver disease (AST or ALT levels greater than 3 times the upper limit of normal or clinical diagnosis)
  - Severe kidney disease (eGFR < 30 ml/min/1.73 m^2^ or clinical diagnosis)
  - Mental health conditions severe enough to impair the ability to provide consent

The current standard of care for patients with OUD in the Center for Healing includes harm-reduction based engagement, MOUD treatment, navigation of care coordination to psychosocial resources, and in-house psychiatric care and counseling for interested patients. The clinic offers two of three FDA-approved medications to treat OUD: buprenorphine (brand names: Suboxone, Subutex, Zubsolv, Sublocade, etc.) and naltrexone (brand name Vivitrol). Buprenorphine is a partial opioid agonist of the Mu and other opioid receptors that reduces or eliminates withdrawal symptoms and reduces cravings [39]. Buprenorphine- containing medications have been shown to decrease opioid use as well as opioid-related morbidity and mortality [40]. Naltrexone, an antagonist, has no agonist activity and instead blocks the effects of opioids [39]. This medication does not treat withdrawal but has been shown to decrease opioid use and cravings [40]. Most patients receiving care at the Center for Healing are being treated for opioid use disorder with evidence-based pharmacotherapy with either buprenorphine or naltrexone though there are a number of patients who are receiving harm reduction interventions only, psychiatric medications, or treatments for other substance use disorders. The majority of patients in the clinic are prescribed a formulation of buprenorphine whether that be Suboxone (buprenorphine-naloxone), Subutex (buprenorphine), or Sublocade (injectable buprenorphine-naloxone). Vivitrol (injectable naltrexone) is only prescribed to a small proportion of patients in the clinic. The clinic offers a mix of individual appointments and group visits that offer peer support. Of note, the clinic has a large perinatal population, including both pregnant and parenting women with substance use disorders, who are referred from throughout South Jersey for the unique care provided that is largely otherwise unavailable to these women.

### Data elements and survey instruments

#### Genetic data

Participants enrolled in the study will consent to genetic analysis up to and including whole genome sequencing, so a variety of genetic assays may be employed to characterize variants in participant DNA. The following planned genetic assays are of particular interest for GOALS:

- Whole genome sequencing to characterize single nucleotide polymorphisms (SNP) that are potentially associated with opioid use phenotypes
- Reduced representation bisulfite sequencing to evaluate DNA methylation variation as a potential risk factors for OUD

#### Non-genetic data

In addition to potential genetic contributions to opioid use and OUD, it is well-documented that non-genetic factors also play a substantial, perhaps primary role in influencing risk for opioid dependence and addiction [41, 42]. With this in mind, we have designed this study to collect extensive environmental information from participants. Clinically validated surveys are implemented to collect information that is known to meaningfully influence risk for OUD, and is not commonly or consistently collected in EHRs. The following surveys are completed by participants at the baseline appointment:

- Addiction Severity Index [38] (ASI-5)
- Adverse Childhood Experiences [37] (ACE)
- Opioid Risk Tool [36] (ORT)
- Patient Health Questionnaire [35] (PHQ-9)
- Generalized Anxiety Disorder [34] (GAD-7)
- Custom survey on perceptions of the role of genetics in OUD

A description of each survey is shown in Table 1. Each of these surveys, with the exception of the ASI, are completed by participants on a tablet computer, aiding confidentiality and potentially reducing the discomfort of sharing personal information with the clinical coordinator. The ASI is conducted as a verbal interview because that is the clinically validated format for the collection of reliable information. The ASI follow-up survey, PHQ-9, and GAD-7 are repeated at 3 additional follow-up appointments at 3 months, 6 months, and 12 months post-study enrollment (Fig. 2).

#### Electronic health records

At the time of consent, participants provide permission for use of their EHR data collected for the duration of their participation in the study. This data is an essential, complementary source of genetic and non-genetic data collected, and provides co-morbid health and treatment outcomes information. Key outcomes include: treatment retention rate, toxicology results, number of overdoses (reported by patients to the research team or documented in EHR), visit frequency (determined by clinic team and inversely correlated with patient clinical stability, with more stable patients visiting less frequently), and goal accomplishments (e.g. changes in employment status, school, family, or housing status as measures of well-being, recorded by clinical team notes in the EHR).

### Data analysis

We plan to employ multivariate regression modeling (for binary and continuous outcomes) to evaluate the relative contributions of genetic and non-genetic factors potentially influencing the development of OUD and related treatment outcomes such as treatment retention rate, percent of urine toxicology results negative for non-prescribed opioids, and overdose events.

We plan to enroll 400 participants. For simple logistic regression modeling of binary outcomes, we have at least 80% power to detect effect size differences (≤ 0.1) between cases and controls when we have a minimum sample size of 400 GOALS cases and 400 opioid exposed controls collected from the OPTIN arm of the study described above; briefly, OPTIN participants are in treatment for chronic pain, may be on provider prescribed opioids, but are not diagnosed with OUD. As sample size decreases, we retain at least 80% power to detect medium and large effect sizes (*n* = 70 in each group, and *n* = 30 in each group, respectively). For simple linear regression modeling of continuous trait outcomes, we have a least 80% power to detect small effect size differences (≤ 0.1) with the full cohort of 400 participants. As sample size decreases, we retain at least 80% power to detect medium and large effect sizes (*n* = 65 and *n* = 30, respectively).

We will incorporate covariates into more complex, multi-variate logistic and linear regression modeling, as appropriate. We will consider demographic covariates such as age, gender, and self-reported race and ethnicity for inclusion in models of genetic and non-genetic factors. In addition, we will consider measures of genetic ancestry for inclusion in models that include genetic factors to offset any potential impact of population stratification. In these analyses, we will evaluate correlation among covariates, and employ approaches to reduce model overfitting (e.g., Akaike’s Information Criterion) [43].

## Discussion

GOALS will extend our understanding of genetic and non-genetic risk factors for OUD and how they contribute to the relative effectiveness of OUD treatments. To our knowledge, this is the first study to integrate, clinically validated survey tools, EHR data, and genomic data.

Clinical studies of participants with substance use disorders are challenging to conduct. The opioid use disorder participant population is vulnerable due to the stigma of OUD, health comorbidities, unstable housing, criminal justice system involvement, and other life disruption associated with substance use disorders [44]. Collecting data from this population is valuable and important. One of the key strengths of this study is the strong commitment of the addiction medicine division within Cooper University Health Care Center for Healing. The clinical team provides specialized training for research staff on substance use disorder to improve language and research engagement within the clinic space. The clinical team also provides the research team with introductions to patients at the clinic. These efforts by the clinical staff build trust and confidence between the research team and the patient community. The results generated from this study will be assessed within the context of currently available treatment modalities to assess whether and how treatment options can be improved in the future.

One limitation of this study is that it does not include patients receiving methadone at the time of enrollment. Patients who enroll in the study could, however, go on to receive methadone later in their treatment course, and referral to opioid treatment programs in the community are common for patients who are not clinically improving on either buprenorphine or naltrexone. Participants who begin receiving methadone after enrollment, can continue to participant in the study. The population is limited to those receiving only two of the three FDA approved medications for opioid use disorder, and is therefore missing treatment outcomes from a key population of patients receiving MOUD as they may interplay with genetic or psychosocial factors. Finally, we note that our power to detect medium and large effect sizes with our target sample size (400 OUD cases and 400 controls) may be reduced by unbalanced covariate population stratification in our regression modeling approach.

### Trial status

Recruitment for this study commenced in March 2019. At the time of submission (June 2020), 85 individuals have been enrolled. Enrollment is expected to continue for 2 years, until March 2021.

## Data Availability

De-identified datasets generated by this study will be made available from the corresponding author on reasonable request following permission guidelines set up in the consent.

## Abbreviations

OUD: Opioid Use Disorder
CDC: Centers for Disease Control and Prevention
GOALS: Genomics of Opioid Addiction Longitudinal Study
CORI: Camden Opioid Research Initiative
OPTIN: Optimizing Pain Treatment in New Jersey
ORT: Opioid Risk Tool
MOUD: Medication for opioid use disorder
EHR: Electronic health records
SNP: Single nucleotide polymorphism
ASI: Addiction Severity Index
ACE: Adverse Childhood Experiences
PHQ-9: Patient Health Questionnaire
GAD-7: Generalized Anxiety Disorder
